# Racial and Ethnic Disparities in Hospitalization and Clinical Outcomes Among Patients with COVID-19

**DOI:** 10.5811/westjem.2022.3.53065

**Published:** 2022-08-11

**Authors:** Felipe Serrano, Erik J. Blutinger, Carmen Vargas-Torres, Saadiyah Bilal, Christopher Counts, Matthew Straight, Michelle P. Lin

**Affiliations:** * Icahn School of Medicine at Mount Sinai, Department of Emergency Medicine, New York, New York; † Icahn School of Medicine at Mount Sinai, Department of Population Health Science and Policy, New York, New York

## Abstract

**Introduction:**

The recent spread of coronavirus disease 2019 (COVID-19) has disproportionately impacted racial and ethnic minority groups; however, the impact of healthcare utilization on outcome disparities remains unexplored. Our study examines racial and ethnic disparities in hospitalization, medication usage, intensive care unit (ICU) admission and in-hospital mortality for COVID-19 patients.

**Methods:**

In this retrospective cohort study, we analyzed data for adult patients within an integrated healthcare system in New York City between February 28–August 28, 2020, who had a lab-confirmed COVID-19 diagnosis. Primary outcome was likelihood of inpatient admission. Secondary outcomes were differences in medication administration, ICU admission, and in-hospital mortality.

**Results:**

Of 4717 adult patients evaluated in the emergency department (ED), 3219 (68.2%) were admitted to an inpatient setting. Black patients were the largest group (29.1%), followed by Hispanic/Latinx (29.0%), White (22.9%), Asian (3.86%), and patients who reported “other” race-ethnicity (19.0%). After adjusting for demographic, clinical factors, time, and hospital site, Hispanic/Latinx patients had a significantly lower adjusted rate of admission compared to White patients (odds ratio [OR] 0.51; 95% confidence interval [CI] 0.34–0.76). Black (OR 0.60; 95% CI 0.43–0.84) and Asian patients (OR 0.47; 95% CI 0.25 – 0.89) were less likely to be admitted to the ICU. We observed higher rates of ICU admission (OR 2.96; 95% CI 1.43–6.15, and OR 1.83; 95% CI 1.26–2.65) and in-hospital mortality (OR 4.38; 95% CI 2.66–7.24; and OR 2.96; 95% CI 2.12–4.14) at two community-based academic affiliate sites relative to the primary academic site.

**Conclusion:**

Non-White patients accounted for a disproportionate share of COVID-19 patients seeking care in the ED but were less likely to be admitted. Hospitals serving the highest proportion of minority patients experienced the worst outcomes, even within an integrated health system with shared resources. Limited capacity during the COVID-19 pandemic likely exacerbated pre-existing health disparities across racial and ethnic minority groups.

## INTRODUCTION

Since the beginning of the coronavirus 2019 (COVID-19) pandemic, over 30.3 million people in the United States have been infected, and over 500,000 have died.^1^ Of these, over 31,000 were in the New York City (NYC) area alone. However, the burden of illness has been unequally distributed among racial and ethnic groups, with early evidence demonstrating substantially higher burden of disease and worse health outcomes among Black, Asian, and Hispanic/Latinx persons.^2^ The disproportionate burden of disease among racial minorities has been consistent with – and has potentially exacerbated – pre-existing disparities in health outcomes.^3^–^10^

The onset of the pandemic in NYC in early 2020 was characterized by an unprecedented surge in the demand for healthcare, associated with limited capacity and resources among healthcare facilities.^11^ One of the most consequential decisions that emergency physicians make on a daily basis is whether to admit a patient to the hospital. The decision to admit became even more challenging when caring for an overwhelming number of patients with a highly communicable disease requiring isolation and limited access to supplemental oxygen and respirators.

While racial and ethnic differences in COVID-19 infection and mortality rates have been well established, the contribution of health systems to these differences in outcomes remains unexplored.^12^–^15^ To date, there has been little research examining differences in emergency department (ED) admission among racial groups and how these differences are associated with health outcomes. Therefore, we examined the association between race and the likelihood of admission among COVID-19 patients presenting to the ED. Among patients admitted to the hospital, we also examined the association of race with the likelihood of medication administration, intensive care unit (ICU) admission, and death.

## METHODS

We conducted a retrospective analysis using electronic health record (EHR) data from a large, urban, academic health system in NYC with three academic sites based in Manhattan and two community-based academic affiliates in Brooklyn and Queens. The institutional review board approved this study. No sponsors or funding were obtained for this study. We adhered to the Strengthening the Reporting of Observational Studies in Epidemiology (STROBE) guidelines for reporting observational studies.

Clinical data for this analysis were extracted from the EHR using an enterprise data warehouse specifically designed to store COVID-19-related patient information. This was performed periodically by a separate group of researchers and shared in a secure, Health Insurance Portability and Accountaility Act-compliant database for ease of analysis by different investigator groups. Data extracted included patient demographics (age, gender, patient-reported race and ethnic group, preferred language, primary expected payer); chronic conditions documented through diagnosis codes in the *International Classification of Disease 10th Revision* (ICD-10); body mass index; detailed visit history such as date(s) of visit, type of visit, initial vital signs, laboratory results, and medications administered. All patients satisfying one or more of the following criteria were included in the database: 1) had a COVID-19 related encounter diagnosis; 2) had an encounter with a COVID-19 related visit type; 3) had an order or result for a severe acute respiratory syndrome coronavirus 2 (SARS-CoV-2) Mount Sinai Health System (MSHS) laboratory test; or 4) had a SARS-CoV-2 test result from the New York State Department of Health (NYSDOH) Wadsworth laboratory.

**Population Health Research Capsule**

What do we already know about this issue?

*COVID-19 has disproportionally affected racial/ethnic minorities. Disparities in treatment have been shown to affect outcomes for numerous other conditions.*

What was the research question?

*Were there racial and ethnic disparities in COVID-19 admission rates from the ED, and in medication administration, and mortality?*

What was the major finding of the study?

*Non-White patients were more likely to seek care for COVID-19 in the ED but had lower adjusted odds of hospital and ICU admission.*

How does this improve population health?

*Lower rates of ED admission among non-White COVID-19 patients after adjusting for clinical severity may be due to structural racism; efforts targeting ED clinicians may reduce disparities.*

Because ethnicity is infrequently reported by non-Hispanic patients and Hispanic/Latinx patients’ race is frequently reported as “other” or unknown, we used a combined race-ethnicity variable derived by the data warehouse as our predictor of interest. Race-ethnicity groups were defined as White, Black, Hispanic/Latinx, Asian, and other (represented by American Indian, other, and unknown). Given that race-ethnicity was our primary variable of interest, we excluded patients with missing race-ethnicity (6.29%). The cohort included adult patients ≥18 years of age.

The primary outcome of interest was admission to the hospital from the ED. Of note, during the COVID-19 pandemic, observation status was suspended due to capacity limitations. For our analysis, we included all patients who had a visit type of ED and/or inpatient and had a positive SARS-CoV-2 test result from the MSHS laboratory or NYSDOH laboratory. Secondary outcomes included likelihood of receiving medications for the treatment of COVID, ICU admission, and in-hospital death. For these outcomes, we also excluded patients discharged from the ED given the reduced likelihood of COVID-19-specific prescription medication administration in outpatient settings. We focused our analysis on medications that were designated primarily for the treatment of COVID-19 to minimize confounders. The primary medications of interest were hydroxychloroquine, remdesivir, interleukin-6 (IL-6) inhibitors as a class (tocilizumab, sarilumab), oral steroids (prednisone, prednisolone, oral dexamethasone), and intravenous (IV) steroids (methylprednisolone, hydrocortisone, IV dexamethasone).

In our primary analysis examining differences in admission rates, we adjusted for the previously listed patient demographics; medical risk factors including obesity, hypertension (classified separately because it is an independent risk factor for poor COVID-19 outcomes but not included in the Charlson Comorbidity Index [CCI] score), smoking status, CCI score,^16^ and abnormal vital signs including oxygen saturation. We also adjusted for hospital site and month, given the differences in resource availability and practice patterns in different hospital settings during different phases of the pandemic. For secondary outcomes among admitted patients, we also adjusted for abnormal laboratory findings (white blood cell including lymphocyte and neutrophil count, platelets, alanine transaminase, troponin, glomerular filtration rate (GFR), D-dimer, C-reactive protein, ferritin, IL-6). We evaluated the inclusion of recently derived but unvalidated COVID-19 risk scores but elected not to include them because they were not available at the time of data collection to emergency clinicians who were making the decision to admit, and due to lack of validation.

We conducted parametric and non-parametric tests for all descriptive statistics, as appropriate. Categorical measures are presented as percentages. Continuous measures are presented as means and standard deviations or medians and interquartile ranges. We conducted bivariate tests of association, and then examined patient-level outcomes using multilevel, multivariable logistic regression to test for differences in the odds of inpatient admission, medication administration, ICU admission, and in-hospital mortality after accounting for hospital-level clustering. We conducted sensitivity analyses by including patients with suspected but not confirmed COVID-19 in the cohort and examining specific medication types in separate models (specifically steroids and hydroxychloroquine). All regression variables were selected a priori. We conducted analyses in Stata 16 (StataCorp LLC, College Station, TX). We adhered to STROBE guidelines for reporting observational studies.

## RESULTS

A total of 4,717 adult patients with a positive SARS-CoV-2 test in the ED or inpatient setting within the time period of interest were included in the primary analysis. Of these, 3,219 (68.2%) were admitted to an inpatient setting and were included in the analyses examining differences in medication administration, ICU admission, and death.

Demographic and clinical data for all patients is by race-ethnicity in Table 1. Black patients were the largest group (29.1%), followed by Hispanic/Latinx (29.0%), White (22.9%), Asian (3.86%), and patients who reported “other” race-ethnicity (15.1%). Hispanic/Latinx patients (27.7%), Asian (25.8%), and Black (18.1%) patients were more frequently insured by Medicaid compared to White patients (7.24%). Black (35.1%), Asian (32.4%), and Hispanic/Latinx (31.8%) patients were also more likely to have the highest chronic disease burden, defined as CCI score 3 or higher compared to White patients (26.6%). Black (33.3%) and Hispanic/Latinx (30%) patients were also more likely to be obese, while Asian (39.6%), Hispanic/Latinx (37.6%), and Black (32.5%) patients were more likely to have been diagnosed with hypertension. Black patients were disproportionately overrepresented at the community-based academic affiliate in Brooklyn, while Asian and Hispanic/Latinx patients were disproportionately overrepresented at the community-based academic affiliate in Queens. Unadjusted mortality was highest among Black (28.2%) and Asian (25.3%) patients.

Table 2 shows characteristics and factors associated with admission from the ED. White patients (24.3%) were disproportionately overrepresented among admitted patients, while Hispanic/Latinx (27.8%) and Black (19.3%) patients were underrepresented. Hispanic/Latinx patients had a significantly lower adjusted rate of admission compared to White patients (odds ratio [OR] 0.51; 95% confidence interval [CI] 0.34–0.76). Patients aged 36–55 years (OR 1.97; 95% CI 1.15–3.40), and 75 years or older (OR1.97; 95% CI 1.02–3.81) were more likely to be admitted relative to those aged 18–35 years. Patients insured by Medicaid (OR 2.02; 95% CI 1.36–2.99) and Medicare (OR 1.63; 95% CI 1.09–2.44) also had significantly higher rates of admission compared to patients with private insurance. Both severe and mild hypoxia were significantly associated with admission (OR 39.6; 95% CI 24.46–64.35 for oxygen saturation (SpO2) 92–96%, and OR 241.7; 95% CI 140.36–416.25 for SpO2 <92%) as was fever (OR 4.59; 95% CI 3.47–6.09). We found significant and progressively higher odds of admission in April through August relative to March. We also found significantly lower odds of admission (OR 0.68; 95% CI 0.46–0.99) at the community-based, academically affiliated hospital site located in Queens compared to the academic, quaternary-care referral hospital of the healthcare system located in Manhattan.

Factors associated with ICU admission are shown in Table 3. Patients of Black (OR 0.60; 95% CI 0.43–0.84) and Asian race (OR 0.47; 95% CI 0.25–0.89) were less likely to be admitted to an ICU setting. Patients in the older age group, over 75 years, (OR 0.4; 95% CI 0.19–0.85) were significantly less likely to be admitted to the ICU relative to those aged 18–35 years. Patients were more likely to be admitted to the ICU if they were obese (OR 1.43; 95% CI 1.12–1.84), severely hypoxic (OR 12.19; 95% CI 1.86–79.78), febrile (OR 1.86; 95% CI 1.23–2.80), or tachypneic (OR 1.92; 95% CI 1.50–2.47) but less likely to be admitted to the ICU if hypotensive (OR 0.26; 95% CI 0.08–0.86). Nearly all lab abnormalities were independently associated with increased ICU admission except for lymphocyte count, D-dimer, and ferritin. Hospital site was again independently associated with outcomes in this analysis with three sites having significantly increased ICU admission relative to the quaternary-care academic hospital, including the community-based academic affiliate sites in Brooklyn (OR 2.96; 95% CI 1.43–6.15) and Queens (OR 1.83; 95% CI 1.26–2.65). Patients were less likely to be admitted to the ICU in April (OR 0.46; 95% CI 0.30–0.49) relative to March.

Table 3 shows factors associated with in-hospital death. Increasing age, tachypnea, hypoxia, elevated troponin, and reduced GFR were independently associated with higher odds of in-hospital mortality. There was significantly higher mortality at the community-based academic affiliate sites in Brooklyn (OR 4.38; 95% CI 2.66–7.24) and Queens (OR 2.96; 95% CI 2.12–4.14) relative to the quaternary-care academic hospital. In-hospital mortality decreased significantly with each time period as the pandemic progressed. Absolute lymphocyte count below 1500 (OR 0.25; 95% CI 0.15–0.44) and receiving hydroxychloroquine (OR 0.58, 95% CI 0.45–0.75) were associated with lower odds of mortality. After adjusting for hospital site, time period, demographics and clinical factors, race was not independently associated with in-hospital mortality.

We also analyzed predictors associated with medication administration. The data is included as Appendix A. Patients were more likely to receive medication if they were severely hypoxic (SpO2 <92%, OR14.18; 95% CI 7.86–25.57), mildly hypoxic (SpO2 92–96%; OR 6.97; 95% CI 3.93–12.35), febrile (OR 1.91; 95% CI 1.52–02.39), treated at the community-based academic affiliate site in Brooklyn (OR 2.53; 95% CI 1.68–3.80), admitted to the ICU (OR 1.42; 95% CI 1.04–1.93) or had abnormal lab values (white cell and lymphocyte count, transaminase, D-dimer, C-reactive protein, ferritin, and IL-6 levels). Patients were less likely to receive medications if they had a CCI score of 3 or higher (OR 0.74; 95% C 0.58–0.94) or were admitted in May through August relative to March. Race, age, insurance, and gender were not associated with odds of receiving COVID-19-related medications.

In sensitivity analyses examining patients with both suspected and confirmed COVID-19, we similarly found significantly lower admission rates among ED patients of Hispanic/Latinx descent although the magnitude of difference was smaller (OR 0.71 vs 0.51). In addition, we found significantly lower rates of medication administration among non-English speaking inpatients; however, this finding did not persist after excluding patients with suspected but unconfirmed COVID-19. When we examined specific types of medications – hydroxychloroquine and steroids separately – we found no differences between racial or ethnic groups.

## DISCUSSION

Black and Hispanic/Latinx patients accounted for the highest proportion of ED patients diagnosed with COVID-19; however, Hispanic/Latinx race-ethnicity was associated with significantly decreased odds of admission compared to White patients. Among hospitalized patients, Black and Asian patients were less likely to be admitted to the ICU relative to White patients. We also observed higher rates of ICU admission and mortality at two community-based, academic affiliate sites serving predominantly Black, Asian, and Hispanic/Latinx populations.

Consistent with prior studies, Black and Hispanic/Latinx patients accounted for the largest proportion of COVID-19 patients and are substantially overrepresented relative to the demographic composition of both NYC and the US.^17^ Despite this, we observed lower rates of inpatient admission for Hispanic/Latinx patients, and lower rates of ICU admission for Black and Asian patients, respectively. The decreased likelihood of admission from the ED for Hispanic/Latinx patients in our analysis contrasts with previous studies that showed either similar or increased odds of admission for this patient population.^2^, ^3^, ^12^, ^13^, ^18^–^20^ The majority of these studies relied on population-level statistics without controlling for disease severity or other demographic characteristics. Our results differ from one recent retrospective analysis out of NYC with similar methods; however, our study sample includes a longer time period and attempts to control for time of presentation and site.^21^ It is possible that in our ED cohort Hispanic/Latinx patients were less sick overall and, therefore, less likely to require admission; however, this is unlikely since we controlled for hypoxia and other clinical indicators of disease severity that are likely to be associated with the decision to admit.

The lower admission rates at the community-based site in Queens may reflect the higher incidence of COVID-19 in that borough and strained capacity relative to Manhattan sites, resulting in fewer available inpatient beds and higher risk discharges.^19^–^21^ Another possible explanation is that Hispanic/Latinx patients were seeking care at hospitals farther from home, given prior research suggesting lower rates of admission are associated with increasing distance to a hospital from a patient’s home. 22 Given that about 75% of frontline workers in NYC are people of color,^23^ it is possible that many Hispanic/Latinx patients in our cohort lived far from the ED they were evaluated in and presented due to the proximity to their place of work. The proportion of Hispanic/Latinx patients (29%) in our sample is similar to that of NYC; however, only two of the top 50 NYC ZIP codes with the highest proportion of Hispanic/Latinx residents are located in close proximity to study sites.^17^ It is possible that these patients would be reluctant to agree to hospitalization given their role as frontline workers and providers for their family.^24^,^25^

The decreased likelihood of admission and ICU utilization among minority patients could have reflected an inherent and systemic bias in our healthcare system toward fewer admissions of minority patients. Prior studies have explored the relationship between race and discriminatory access to healthcare resources, leading to decreased healthcare utilization among minority populations due to the expectation of worse outcomes or inability to pay.^26^,^27^ Hispanic/Latinx and Asian patients may also not have been able to communicate the severity of their symptoms due to language barriers. Our findings raise concerns that in the resource-depleted setting of a major pandemic, allocation of limited inpatient and ICU beds may have been racially biased.

Even within our healthcare system with a shared resource pool, we found significant differences in admission rates and clinical outcomes between different hospital sites, specifically higher rates of ICU admission and in-hospital mortality at two community-based, academic affiliates serving a disproportionately higher share of Black (Brooklyn), Asian and Hispanic/Latinx (Queens) patients. Prior research has demonstrated poor COVID-19 outcomes associated with different settings of care.^28^ Not only are Black, Asian, and Hispanic/Latinx patients more likely to have pre-existing conditions and lack health insurance, leading to increased morbidity and decreased access to care,^29^ they are also more likely to live in racially segregated neighborhoods 30 and seek care at minority-serving hospitals, which are often less well-funded and have fewer licensed and ICU beds per inhabitant.^31^ Prior evidence on maternal mortality has identified even wider disparities among Black patients treated at facilities primarily serving Black patients.^32^

Additionally, Black patients in our cohort were more likely to be female compared to their White counterparts. Studies have shown that male gender is associated with increased COVID-19 mortality,^33^ which suggests Black male patients were less likely to present to the ED when ill with COVID-19. This may reflect distrust of the medical system, decreased access to care or, as described above, decreased willingness to seek care given financial difficulties. While we did not examine within-hospital site differences in outcomes by race, further analysis of disparities in COVID-19 outcomes after accounting for community demographics is needed.

Our findings confirm that age is an independent risk factor for inpatient mortality, even after adjusting for other markers of pre-existing disease, severity of illness or demographics, consistent with prior studies.^34^–^36^ However, we found decreasing odds of being admitted to the ICU with increasing age. Combined with the increased odds of admission over time, these findings suggest allocation based on likelihood of survival due to extremely limited resources during a pandemic. Additional research is needed to further examine the impact of non-clinical factors on clinical care and resource allocation in a pandemic situation.

## LIMITATIONS

Our analysis has several limitations. Our population was limited to one integrated healthcare system in NYC, which limits its generalizability to other settings; however, the study sample is diverse, and our findings are consistent with several national studies identifying disparities in utilization and outcomes. Our findings are also limited by the exclusion of the 6% of patients with missing data for race and ethnicity. While missing race-ethnicity data may be non-random, using imputation methods to estimate the probability of belonging to different racial groups, such as geocoding or surname data, only produces probability of belonging to a certain group, which may have underestimated their sampling variability and led to bias in our analysis.^37^

In addition, as described above, we did not have data on neighborhood, socioeconomic status, and other social determinants, which limits our ability to draw conclusions regarding geolocalized economic and racial factors that may be contributing to differential admission rates and clinical outcomes in our patient population. Lastly, we did not have data on endotracheal intubation or ventilation, given that our analysis was limited to structured data fields readily extracted from the EHR, and procedures such as intubation are more likely to appear in clinician documentation or billing records. However, we examined other patient-centered outcomes including ICU admission and death and adjusted for numerous clinical indicators of severity of illness.

## CONCLUSION

In this largely diverse, urban, and multicultural population, we found a disproportionate burden of disease and disparities in care among minority populations that was likely exacerbated by limited resources during the COVID-19 pandemic. Non-White patients accounted for a disproportionate share of COVID-19 patients seeking care in the ED but were less likely to be admitted to the ICU or hospitalized. Furthermore, hospitals serving the highest proportion of minority patients experienced the worst outcomes, even within an integrated health system serving a diverse patient population. Dismantling structural racism within the healthcare system and our society as a whole is necessary to improve the health and well-being of historically marginalized populations.

## Supplementary Information



## Figures and Tables

**Table 1:** Characteristics of study sample by race and ethnicity.

Patient/hospital characteristic	Total N = 4,717	White (22.85%, N = 1,078)	Black (29.13%, N = 1,374	Hispanic/Latinx (29.02%, N = 1,369)	Asian (3.86 %, N = 182)	Other (15.14%, N = 714)
Age, mean (SD)	63.51 (17.33)	68.70 (17.38)	61.56 (16.54)	61.90 (17.74)	63.22 (15.92)	62.61 (16.81)
Female	46.92	42.95	51.75	47.48	40.66	44.12
English as primary language	80.33	91.47	96.51	54.93	74.73	82.49
Insurance						
Missing	1.08	1.21	1.02	1.02	1.65	0.98
Medicaid	19.31	7.24	18.05	27.68	25.82	22.27
Medicare	48.95	62.15	44.76	46.38	37.36	44.96
Private	22.28	22.36	27.80	16.07	28.02	21.99
Other	7.29	6.03	7.50	7.38	7.14	8.68
Self-pay	1.08	1.02	0.87	1.46	0.00	1.12
Hospital Site						
Brooklyn	21.05	29.22	36.24	3.51	20.33	13.31
Queens	17.77	18.27	6.19	25.20	37.36	20.03
Manhattan 1	32.99	29.87	28.31	35.87	25.82	43.00
Manhattan 2	9.24	14.94	6.11	8.84	10.44	7.14
Manhattan 3	18.95	7.70	23.14	26.59	6.04	16.53
Time period (2020)						
March 1–March 31	42.61	42.67	44.54	41.49	37.36	42.30
April 1–30	46.24	45.45	43.81	47.48	51.10	48.46
May 1–31	6.21	6.77	7.13	5.33	7.69	4.90
June 1–Aug 19	4.94	5.10	4.51	5.70	3.85	4.34
Total prior visits *						
0	97.46	97.59	97.02	97.22	95.05	99.16
1	2.40	2.13	2.84	2.63	4.95	0.84
2+	0.15	0.28	0.15	0.15	0.00	0.00
Past Medical History						
Hypertension	32.65	28.48	32.53	37.62	39.56	27.87
CCI score 0	58.30	58.72	58.15	54.57	58.24	65.13
CCI score 1–2	11.30	14.66	10.04	10.30	9.34	11.06
CCI score 3+	30.40	26.62	31.80	35.14	32.42	23.81
Obesity (BMI ≥30)	28.47	24.86	33.26	29.95	12.64	25.91
Smoker (active/former/intermittent)	28.58	29.31	30.35	28.12	26.92	25.35
Initial Vital Signs						
Temperature ≥37.5° Celsius	65.06	60.58	62.66	67.86	69.78	69.89
Heart rate ≥90	64.83	58.44	65.72	68.01	62.64	67.23
Respiratory rate ≥22	31.21	29.59	27.44	33.46	34.07	35.85
Systolic BP ≤100	1.95	2.32	1.67	1.61	1.65	2.66
SpO_2_ ≥96%	20.27	16.79	25.18	18.41	20.88	19.47
SpO_2_ 92–95%	27.54	26.72	27.80	29.00	17.03	28.15
SpO_2_ <92%	52.02	56.40	46.72	52.52	62.09	52.10
Initial Lab Tests						
White blood cell count <4K or >12K	23.81	24.77	16.74	25.86	32.97	29.69
Absolute neutrophil count <500	18.00	17.44	14.56	19.72	24.18	20.59
Absolute lymphocyte count <1500	81.03	82.00	78.17	82.25	80.22	82.91
Platelet count	2.97	2.23	2.69	3.58	80.22	3.78
<1500 per mm^3^	2.97	2.23	2.69	3.58	1.65	3.78
ALT ≥40 U/L	42.02	39.98	37.19	44.85	52.75	46.22
Troponin ≥0.04 pg/L	53.17	54.36	53.28	51.79	56.04	53.06
GFR 15–60 ml/min	43.80	46.29	46.43	39.08	43.96	43.98
GFR <15 ml/min	15.35	11.78	19.58	13.59	16.48	15.69
D-dimer ≥0.5 mg/L	59.55	58.44	56.55	61.50	60.99	62.89
CRP ≥16.6 mg/L	25.61	25.88	30.79	24.03	19.23	19.89
Ferritin >300 μg/L	52.51	51.86	48.98	52.59	60.99	57.98
IL-6 ≥ 80 pg/mL	19.19	17.81	18.56	18.63	24.18	22.27
Medications (% receiving)						
Any medication	52.70	53.90	51.38	52.30	55.49	53.50
Hydroxychloroquine	46.83	47.31	46.00	46.53	49.45	47.62
Remdesivir	1.65	1.86	1.31	1.75	2.20	1.68
IL-6 inhibitor	3.52	3.25	4.00	2.56	7.69	3.78
Steroids (PO + IV)	20.75	19.67	17.47	23.16	28.02	22.27
Outcomes						
Hospital days, median	6.92 (3.87 – 11.89)	6.83 (3.94 – 11.82)	7.12 (4.08 – 12.51)	6.87 (3.62 – 11.11)	8.02 (4.33 13.24)	6.41 (3.49 11.61)
ICU admission	14.54	14.29	13.25	15.41	13.19	16.11
ICU days, median (IQR)	4.43 (1.85 – 9.59)	3.73 (1.80 – 7.68)	4.85 (2.03 – 10.36)	4.61 (1.75 – 11.16)	6.80 (1.72 13.21)	3.89 (1.90 9.42)
Died in hospital %	22.56	28.20	19.36	19.87	25.27	24.65

* Total prior encounters ≤14 days before index ED encounter (all encounter types, including outpatient and telehealth).

*SD*, standard deviation; *CCI*, Charlson comorbity index; *BMI*, body mass index; *BP*, blood pressure; *SpO_2_*, oxygen saturation; *ALT*, alanine transaminase; *GFR*, glomerular filtration rate; *CRP*, C-reactive protein; *IV*, intravenous; *ICU*, intensive care unit; *U/L*, units per liter; *μg/L*, micrograms per liter; *pg/L*, picogram per liter; *IL-6*, interleukin-6; *PO*, by mouth; *IQR*, interquartile range.

**Table 2:** Characteristics and factors associated with emergency department admission.

Characteristic	Total N = 4,717	Admitted (68.24%) N = 3,219	Discharged (31.76%) N = 1,498	Odds ratio for admission	95% Confidence interval
Age Groups (%)					
1 (18–35)	8.14	3.39	18.36	Ref	-
2 (36–55)	21.94	16.74	33.11	1.97*	1.15 – 3.40
3 (56–65)	20.92	20.35	22.16	1.24	0.72 – 2.13
4 (66–75)	22.3	25.54	15.35	1.70	3.13 – 3.13
5 (76+)	26.69	33.99	11.01	1.97*	1.02 – 3.81
Female	46.92	44.77	51.54	1.15	0.88 – 1.50
English as primary language	80.33	78.60	84.05	0.92	0.63 – 1.34
Race					
White (reference)	22.85	24.29	19.76	Ref	-
Black	29.13	27.80	31.98	0.74	0.50 – 1.10
Hispanic/Latinx	29.02	28.64	29.84	0.51**	0.34 – 0.76
Asian	3.86	4.01	3.54	0.74	0.39 – 1.43
Other	15.14	15.25	14.89	0.77	0.47 – 1.25
Insurance					
Missing	1.08	0.75	1.80	0.61	0.19 – 1.96
Medicaid	19.31	19.20	19.56	2.02**	1.36 – 2.99
Medicare	48.95	59.89	25.43	1.63*	1.09 –2.44
Private (reference)	22.28	13.23	41.72	Ref	-
Other	7.29	6.77	8.41	1.64	0.89 – 3.02
Self-pay	1.08	0.16	3.07	0.55	0.12 –2.56
Hospital Site					
Brooklyn	21.05	15.81	32.31	1.17	0.77 – 1.78
Queens	17.77	19.07	14.95	0.68*	0.46 – 0.99
Manhattan 1	32.99	34.54	29.64	Ref	-
Manhattan 2	9.24	10.07	7.48	0.66	0.43 – 1.01
Manhattan 3	18.95	20.50	15.62	0.86	0.59 –1.25
TIME PERIOD					
March 1–March 31	42.61	34.86	59.28	Reference	-
April 1–30	46.24	53.25	31.17	1.92**	1.45 – 2.54
May 1–31	6.21	7.52	3.40	5.71**	2.99 – 10.90
June 1–Aug 19	4.94	4.38	6.14	7.40**	3.80 – 14.42
Prior visits***					
0	97.46	97.17	98.06	Reference	
1	2.40	2.67	1.80	0.85	0.37 – 1.94
2+	0.15	0.16	0.13	3.01	0.79 – 11.50
Past Medical History					
Hypertension	32.65	38.83	19.36	0.95	0.70 – 1.30
CCI score 0	58.30	49.74	76.70	Reference	
CCI score 1–2	11.30	13.58	6.41	1.48	0.99 – 2.22
CCI score 3+	30.40	36.69	16.89	1.35	0.98 – 1.86
Obesity (BMI ≥30 kg/m^2^)	28.47	32.68	19.43	0.79	0.60 – 1.05
Smoker (Active/former intermittent)	28.58	31.62	22.03	0.93	0.70 –01.22
Initial Vital Signs					
Temperature ≥37.5°C	65.06	79.93	33.11	4.59	3.47 – 6.09
Heart rate ≥90	64.83	68.47	57.01	1.02	0.77 – 1.34
Respiratory rate ≥22	31.21	41.35	9.41	1.31	0.94 – 1.82
Systolic BP ≤100	1.95	1.93	2.00	0.50	0.22 – 1.12
SpO_2_ ≥96%	20.27	1.12	61.42	Reference	
SpO_2_ 92–95%	27.54	26.75	29.24	39.67**	24.46 – 64.35
SpO_2_ <92%	52.02	72.13	8.81	241.71**	140.36 – 416.25

* P <0.05;

** P <0.01.

*** Total prior encounters ≤14 days before index ED encounter (all encounter types, including outpatient and telehealth).

*CCI*, Charlson comorbidity index.

*BMI*, body mass index; *BP*, blood pressure; *C*, Celsius; *SpO2*, oxygen saturation.

**Table 3:** Characteristics and factors associated with intensive care unit admission and in-hospital mortality.

Characteristic	Admitted to ICU (21.31%) N = 686	Not admitted to ICU (78.69 %) N = 2,533	Odds ratio for ICU admission	95% Confidence interval	Died (29.89%) N = 962	Survived (70.11%) N = 2,257	Odds ratio for death	95% Confidence interval
Age Groups %								
18–35 years	4.23	8.81	Reference	-	0.19	10.46	Reference	-
36–55 years	18.37	22.55	0.70	0.34 – 1.45	7.71	26.09	9.25*	1.26 – 67.75
56–65 years	22.01	20.74	0.59	0.29 – 1.22	15.51	22.50	16.31**	2.23 – 119.15
66–75 years	29.30	21.11	0.61	0.29 – 1.28	25.09	21.49	20.42**	2.75 – 151.72
76+ years	26.09	26.79	0.40*	0.19 – 0.85	51.50	19.46	51.75**	6.96 – 384.74
Female	40.23	45.99	1.03	0.81 – 1.31	44.49	44.88	0.89	0.72 – 1.12
English as primary language	76.53	79.16	0.79	0.57 – 1.08	75.78	79.80	0.94	0.70 – 1.27
Race								
White (reference)	22.45	22.92	Reference	-	28.57	21.19	Reference	-
Black	26.53	29.57	0.60**	0.43 – 0.84	25.00	30.33	0.76	0.56 – 1.02
Hispanic/Latinx	30.76	28.73	0.82	0.57 – 1.17	25.56	30.03	0.80	0.58 – 1.11
Asian	3.50	3.92	0.47*	0.25 – 0.89	4.32	3.72	0.62	0.35 – 1.11
Other	16.76	14.86	0.77	0.52 – 1.13	16.54	14.73	1.02	0.71 – 1.44
Insurance				-				
Missing	1.60	0.51	2.93	0.69 – 12.45	0.42	0.89	1.29	0.16 – 10.59
Medicaid	20.55	18.83	1.07	0.72 – 1.58	12.79	21.93	1.42	0.92 – 2.21
Medicare	55.69	61.03	0.70	0.48 – 1.03	74.01	53.88	1.30	0.88 – 1.92
Private	13.99	13.03	Ref	-	7.38	15.73	Ref	-
Other	8.02	6.44	1.01	0.59 – 1.73	5.41	7.35	1.37	0.80 – 2.34
Self-pay	0.15	0.16	-	-	-	0.22	-	-
Hospital Site				-				
Brooklyn	13.56	16.42	2.96**	1.43 – 6.15	20.69	13.74	4.38**	2.66 – 7.24
Queens	16.76	19.70	1.83**	1.26 – 2.65	23.80	17.06	2.96**	2.12 – 4.14
Manhattan 1	35.86	34.19	Reference	-	28.27	37.22	Reference	
Manhattan 2	12.24	9.47	2.62*	1.18 – 5.84	7.38	11.21	1.06	0.58 – 1.93
Manhattan 3	21.57	20.21	1.71	0.79 – 3.70	19.85	20.78	1.52	0.86 – 2.70
Time period				-				
March1–March 31	46.36	31.74	Reference	-	38.15	33.45	Reference	-
April 1–30	40.38	56.73	0.38**	0.30 – 0.49	55.72	52.19	0.81	0.63 – 1.05
May 1–31	8.16	7.34	0.90	0.56 – 1.46	5.09	8.55	0.41**	0.24 – 0.70
June 1–Aug 19	5.10	4.18	1.88	0.92 – 3.85	1.04	5.80	0.08**	0.03 – 0.24
Total prior visits***				-				
0	96.79	97.28	Reference	-	97.51	97.03	Reference	
1	2.77	2.65	1.09	0.56 – 2.12	2.29	2.84	0.86	0.45 – 1.62
2+	0.44	0.08	4.83	0.96 – 24.33	0.21	0.13	0.69	0.14 – 3.30
Past medical				-				
Hypertension	37.76	39.12	0.79	0.61 – 1.03	42.52	37.26	1.06	0.83 – 1.35
CCI score 0	47.38	50.38	Ref	-	46.47	51.13	Ref	-
CCI score 1–2	90.00	13.70	0.98	0.67 – 1.41	14.97	12.98	1.06	0.77 – 1.47
CCI score 3+	271.00	35.93	1.00	0.75 – 1.33	38.57	35.89	1.00	0.77 – 1.30
Obesity (BMI ≥30 kg/m^2^)	40.52	30.56	1.43**	1.12 – 1.84	30.15	33.76	1.10	0.88 – 1.39
Smoker (active/former intermittent)	32.65	30.56	1.12	0.88 – 1.44	32.33	31.32	1.06	0.84 – 1.33
Initial vital signs				-				
Temperature ≥ 37.5 °Celsius	91.98	76.67	1.86**	1.23 – 2.80	83.26	78.51	0.82	0.61 – 1.10
Heart rate ≥ 90	74.20	66.92	1.19	0.91 – 1.55	68.81	68.32	1.11	0.88 – 1.40
Respiratory rate ≥22	59.33	36.48	1.92**	1.50 – 2.47	54.78	35.62	1.66**	1.32 – 2.10
Systolic BP ≤100	1.02	2.17	0.26	0.08 – 0.86	2.70	1.60	1.25	0.61 – 2.55
SpO_2_ ≥96%	0.29	1.34	Ref		0.21	1.51	Ref	
SpO_2_ 92–95%	8.60	31.66	5.04	0.76 – 33.35	6.03	35.58	1.06	0.48 – 2.36
SpO_2_ <92%	91.11	67.00	12.19**	1.86 – 79.78	93.76	62.92	5.33**	2.45 – 11.61
Initial Lab Tests				-				
White blood cell count < 4K or >12K	44.61	28.62	2.18**	1.46 – 3.25	39.09	29.02	1.23	0.87 – 1.74
Absolute neutrophil count <500	37.76	21.63	1.61**	1.15 – 2.27	32.43	21.93	1.02	0.74 – 1.42
Absolute lymphocyte count <1500	98.25	97.79	1.74	0.79 – 3.83	96.15	98.63	0.25**	0.15 – 0.44
Platelet count < 1500 per mm^3^	9.62	2.76	2.06**	1.29 – 3.30	5.72	3.59	0.95	0.54 – 1.68
ALT ≥40 U/L	73.47	51.52	1.46**	1.13 – 1.88	62.47	53.52	0.93	0.73 – 1.18
Troponin ≥0.04 pg/L	78.86	65.06	1.54**	1.17 – 2.03	78.38	63.58	1.53**	1.19 – 1.97
GFR 15–60 ml/min	76.82	51.80	2.81**	2.15 – 3.68	82.22	46.43	2.84**	2.22 – 3.64
GFR <15 ml/min	39.36	16.78	2.36**	1.80 – 3.08	40.33	13.60	3.02**	2.31 – 3.94
D-dimer ≥0.5 mg/L	90.52	77.62	1.56*	1.01 – 2.41	84.82	78.47	0.94	0.65 – 1.36
CRP ≥16.6 mg/L	42.71	32.37	1.87*	1.00 – 3.48	38.88	32.74	0.98	0.61 – 1.59
Ferritin >300 μg/L	84.55	68.73	0.77	0.53 – 1.11	81.08	68.28	1.19	0.86 – 1.64
IL-6 ≥80 pg/mL	57.00	19.62	3.92**	3.02 – 5.08	43.56	20.78	1.94**	1.48 – 2.53
Outcomes								
Hospital days, median (IQR) ****	10.52 (5.26– 20.63)	6.35 (3.64–10.49)	-	-	77.86	73.24	-	-
Died in hospital	54.96	23.10	-	-	67.67	66.59	0.58**	0.45 – 0.75

* P <0.05;

** P <0.01.

*** Total prior encounters ≤14 days before index ED encounter (all encounter types, including outpatient and telehealth).

*** Frequencies reported but excluded from the model as length of stay is confounded by mortality.

*ICU*, intensive care unit; *CCI*, Charlson comorbidity index; *BMI*, body mass index; *BP*, blood pressure; *SpO_2_*, oxygen saturation; *k*, thousand; *mm^3^*, millimeters cubic; *U/L*, units per liter; *mg/L*, milligrams per liter; *μg/L*, micrograms per liter; *pg/mL*, picograms per milliliter; *ALT*, alanine transaminase; *GFR*, glomerular filtration rate; *CRP*, C-reactive protein; *IQR*, interquartile range.
