# Supplementary material for: Racial and Ethnic Disparities in Hospitalization and Clinical Outcomes Among Patients with COVID-19
Source: West J Emerg Med. 2022 Aug 11;23(5):601–12. doi: 10.5811/westjem.2022.3.53065 (PMC9541974; doi:10.5811/westjem.2022.3.53065)
Supplement: Supplementary file 1 [file wjem-23-601-s001.docx]

**Appendix A:** Characteristics and Factors Associated with Medication Administration.

| **Characteristic** | **Received meds (74.62%)**  **N= 2,402** | **Did not receive meds (25.38%)**  **N= 817** | **Odds Ratio for Receiving Medication** | **95% Confidence Interval** |
| --- | --- | --- | --- | --- |
| Age Groups. % |  |  |  |  |
| 1 (18-35) | 3.02 | 13.85 | Reference |  |
| 2 (36-55) | 17.34 | 27.07 | 1.48 | 0.90 - 2.44 |
| 3 (56-65) | 21.00 | 20.84 | 1.13 | 0.69 - 1.86 |
| 4 (66-75) | 26.67 | 17.44 | 1.06 | 0.62 - 1.81 |
| 5 (76+) | 31.98 | 20.80 | 0.86 | 0.50 - 1.49 |
| Female | 43.80 | 47.61 | 1.03 | 0.84 -01.26 |
| English as primary language | 78.48 | 78.95 | 0.82 | 0.63 - 1.06 |
| RACE |  |  |  |  |
| White | 23.37 | 22.28 | Reference |  |
| Black | 28.40 | 29.94 | 1.06 | 0.79 - 1.41 |
| Hispanic/Latinx | 28.80 | 29.27 | 0.84 | 0.63 - 1.11 |
| Asian | 4.06 | 3.63 | 0.90 | 0.54 - 1.51 |
| Other | 15.37 | 14.88 | 0.87 | 0.62 - 1.22 |
| **Insurance** |  |  |  |  |
| Medicaid | 18.15 | 182.00 | 1.03 | 0.75 - 1.43 |
| Medicare | 60.28 | 480.00 | 1.19 | 0.85 - 1.67 |
| Private | 13.95 | 91.00 | Reference |  |
| Other | 7.16 | 46.00 | 0.80 | 0.51 - 1.26 |
| Self-pay | 0.12 | 2.00 | 2.20 | 0.34 - 14.38 |
| **Hospital Site** |  |  |  |  |
| Brooklyn | 15.82 | 15.79 | 2.53** | 1.68 - 3.80 |
| Queens | 17.82 | 22.77 | 0.99 | 0.74 - 1.33 |
| Manhattan 1 | 35.43 | 31.95 | Reference |  |
| Manhattan 2 | 9.87 | 10.65 | 0.82 | 0.52 - 1.31 |
| Manhattan 3 | 21.07 | 18.85 | 0.94 | 0.61 - 1.44 |
| **TIME PERIOD** |  |  |  |  |
| March1-March31 | 38.05 | 25.46 | Reference |  |
| April 1-30 | 56.66 | 43.21 | 0.82 | 0.65 - 1.04 |
| May 1-31 | 3.91 | 18.12 | 0.17** | 0.11 - 0.25 |
| June 1-Aug19 | 1.37 | 13.22 | 0.17** | 0.09 -0.31 |
| **Total prior visits*** |  |  |  |  |
| 0 | 97.17 | 97.18 | Reference |  |
| 1 | 2.66 | 2.69 | 0.99 | 0.60 - 1.62 |
| 2+ | 0.17 | 0.12 | 0.74 | 0.15 - 3.58 |
| **Past Medical History** |  |  |  |  |
| Hypertension | 37.64 | 42.35 | 0.88 | 0.70 - 1.11 |
| Charlson Score 0 | 52.12 | 42.72 | Ref | - |
| Charlson Score 1-2 | 13.24 | 14.57 | 0.88 | 0.64 - 1.19 |
| Charlson Score 3+ | 34.64 | 42.72 | 0.74* | 0.58 - 0.94 |
| Obesity (BMI >=30) | 34.76 | 26.56 | 1.20 | 0.98 - 1.48 |
| Smoking (Active/ Former Intermittent) | 31.89 | 30.84 | 1.11 | 0.90 - 1.37 |
| **Initial Vital Signs** |  |  |  |  |
| Temperature ≥ 37.5 C | 85.30 | 64.14 | 1.91** | 1.52 -02.39 |
| Heart Rate ≥ 90 | 69.86 | 64.38 | 0.99 | 0.80 -01.22 |
| Respiratory Rate ≥22 | 43.80 | 34.15 | 1.02 | 0.82 -01.28 |
| Systolic BP ≤100 | 1.29 | 3.79 | 0.61 | 0.32 -01.17 |
| SpO2 ≥ 96% | 0.37 | 3.30 | Reference |  |
| SpO2 92-96% | 20.86 | 44.06 | 6.97** | 3.93 -012.35 |
| SpO2 < 92% | 78.77 | 52.63 | 14.18** | 7.86 -025.57 |
| **Initial Lab Tests** |  |  |  |  |
| White blood cell count < 4k or >12k | 33.60 | 27.42 | 1.51** | 1.15 -01.98 |
| Absolute neutrophil count < 500 | 26.64 | 20.44 | 1.27 | 0.94 -01.72 |
| Absolute lymphocyte count < 1500 | 98.67 | 95.59 | 4.38** | 2.59 -07.40 |
| Platelet count > 1500 per mm3 | 4.75 | 2.69 | 1.40 | 0.75 -02.63 |
| ALT ≥ 40 U/L | 61.32 | 41.13 | 1.35* | 1.09 -01.66 |
| Troponin ≥ 0.04 | 70.23 | 61.44 | 1.07 | 0.86 -01.33 |
| GFR 15-60 | 58.70 | 52.51 | 0.98 | 0.79 -01.21 |
| GFR <15 | 21.94 | 20.56 | 0.79 | 0.59 -01.04 |
| D-Dimer ≥ 0.5 mg/L | 85.76 | 64.50 | 2.33** | 1.75 -03.09 |
| CRP ≥ 16.6 mg/L | 38.38 | 23.38 | 1.70* | 1.17 -02.49 |
| Ferritin > 300 μg/L | 78.39 | 53.61 | 1.56** | 1.22 -01.99 |
| IL-6 ≥ 80 pg/mL | 32.93 | 11.87 | 1.94** | 1.44 -02.59 |
| **OUTCOMES** |  |  |  |  |
| **ICU Admission** | 24.15 | 12.97 | 1.42* | 1.04 -01.93 |
| Hospital days, median (IQR)**** | 7.52 ( 4.24 12.79) | 4.95 (2.55 9.17) |  |  |
| ICU admission**** | 24.15 | 12.97 |  |  |
| Died in hospital**** | 31.18 | 26.07 |  |  |

*P<0.05

**P<0.01

***Total prior encounters <=14 days before index ED encounter (all encounter types, including outpatient and telehealth)

****Descriptive statistics reported but not included in model as these outcomes are more likely to be dependent on medication administration; however, ICU admission may be associated with likelihood of medication administration
